# Global habitat suitability modeling reveals insufficient habitat protection for mangrove crabs

**DOI:** 10.1038/s41598-022-26226-7

**Published:** 2022-12-15

**Authors:** Masoud Yousefi, Reza Naderloo

**Affiliations:** School of Biology, College of Science, University of Tehran, Tehran, 14155-6455 Iran

**Keywords:** Biodiversity, Biogeography, Biooceanography, Ecological modelling

## Abstract

Mangrove crabs are important components of mangrove forests however their large scale habitat suitability and conservation received little attention. The *Metopograpsus thukuhar*/*cannicci* species complex is a mangrove dwelling species occurs in the Indo-Pacific mangrove forests. Since identifying the complex suitable habitat is critical for its conservation, we modeled global habitat suitability of the complex within marine biogeographic realms and estimated representation of the complex suitable habitats within marine protected areas. We found that the complex’ largest and smallest suitable ranges are located in Central Indo-Pacific and Temperate Southern Africa realms, respectively. Only 12.5% of the complex suitable habitat is protected. The highest proportion of the complex’ protected suitable habitat (22.9%) is located in Western Indo-Pacific realm while the lowest proportion of the complex’ protected suitable habitat (1.38%) is located in Central Indo-Pacific realm. Suitable unprotected habitats of the complex identified in this study have high priority for conservation and should be included in marine protected areas to ensure species conservation. Our results show that species distribution models are practical tools to study marine species distribution across large spatial scales and help marine conservation planning.

## Introduction

Mangrove forests occur in the tropics and subtropics and are among the most threatened ecosystems in the world^1–3^. These highly productive ecosystems cover 167,000 km^2^ and span more than 120 countries and territories^1,2^. Mangroves provide important ecological and economical ecosystem services such as preventing erosion, acting as natural barriers for floods, storms and cyclones, contributing to global climate change mitigation, providing habitat and food for migratory birds, fish, molluscs and crustaceans^2,4–9^. In many countries indigenous people used mangrove biota like crabs and fishes as food^10^.

Despite their ecological importance and ecosystem services they provide, mangroves have been lost at a rate of 0.39% per year since 2000^6^ and are threatened by aquaculture activities, coastal development, climate change and sea level rise^2,11–19^. More importantly mangroves have received much less attention compared to other tropical ecosystems like rain forests and coral reefs and small proportion of them are legally under protection^1,20^.

Mangrove crabs are important components of mangrove forests^21,22^. Mangrove crabs’ richness reaches a peak in Indian Ocean and West Pacific Ocean. Sea surface temperature, nitrate, calcite and dissolved oxygen are important drivers of mangrove crabs’ latitudinal diversity^23^. For effective management of mangrove crabs and consequently mangrove ecosystems, it is important to identify individual mangrove-dwelling species suitable habitat and ecological determinants of their distribution. Besides, it is not well assessed whether marine protected areas are effective in conservation of mangrove crabs. In this regard, Species Distribution Models (SDMs) are practical tools as they are frequently being applied in studying species biogeography, ecology and conservation^24–26^.

Species Distribution Models need species occurrence records and environmental variables to estimate target species probability of presence in a user defined geographic region^24,27^. These models are used in predicting species distribution^28–33^, identifying environmental derivers of species distribution^28,32–34^ assessing protected area effectiveness in conservation of species^35–37^ and quantifying the impacts of future climatic changes on species distribution^38–45^. For instance, Luan et al.^28^ applied SDMs and modeled the spatial distribution of three portunid crabs (*Charybdis bimaculata*, *Charybdis japonica* and *Portunus trituberculatus*) in China. They showed that sea bottom temperature, sea bottom salinity and sediment type were the most important factors affecting the crabs’ distribution. In another study, Compton et al.^35^, used SDMs and modeled potential distribution of the European green crab (*Carcinus maenas*) to identify potential areas of invasion by this highly adaptable estuarine crab.

The Indo-Pacific genus *Metopograpsus* of the family Grapsidae comprises six recognised species including *Metopograpsus thukuhar* which is a mangrove-dwelling species^46–48^. According to the most recent taxonomic account on the genus *Metopograpsus*, *M. thukuhar* is species complex and there are two pseudo-cryptic species in the Indian and the Pacific oceans^48^. *Metopograpsus thukuhar* distributed in the eastern Indian and Pacific oceans, while *M. cannicci*, occurs in the Red Sea, East African coast (from Somalia to Mozambique), Seychelles; Dar es Salaam, Madagascar, Toliara and Toamasina, Mauritius and Persian Gulf and Gulf of Oman^47,48^. Both species (hereafter *Metopograpsus thukuhar*/*cannicci* species complex) live in high density within the trunks and pneumatophores of mangroves, adults mostly seen in natural crevices and juveniles commonly found on surface^47^. The medium-sized species is considered omnivorous, but principally feeding on the macroalgae and mangroves leaves, while rarely preying on smaller crabs^49^. The species occupy mangrove ecosystem with similar ecological conditions but the complex large scale habitat suitability and conservation received little attention.

While, knowledge on the complex habitat suitability and representation of the species suitable habitats within protected areas is necessary for the complex conservation. Thus, the aims of the present study are to predict habitat suitability of the *M. thukuhar*/*cannicci* species complex using the Maximum Entropy (MaxEnt) algorithm, identify the most influential factors in shaping the complex distribution and estimate marine protected area coverage for the complex suitable habitats.

## Results

**Figure 1:**
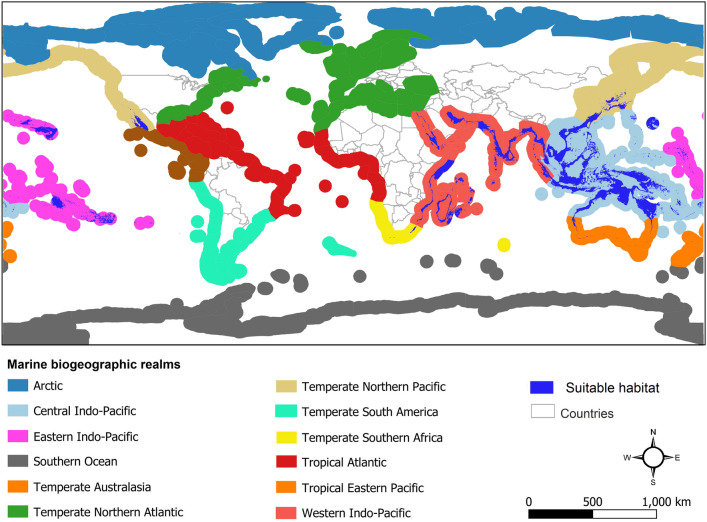
Habitat suitability of the *Metopograpsus thukuhar*/*cannicci* species complex in marine biogeographic realms. Map was generated using QGIS 3.4.1 (https://www.qgis.org).

**Figure 2:**
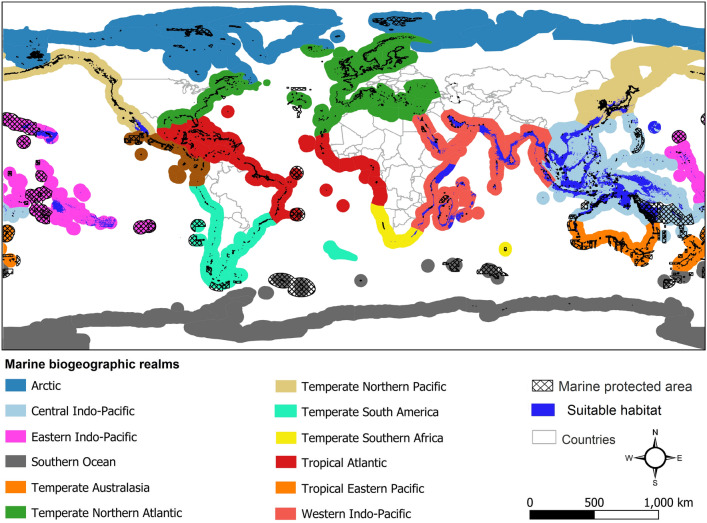
Protected areas coverage for suitable habitats of the *Metopograpsus thukuhar*/*cannicci* species complex based on marine biogeographic realms. Map was generated using QGIS 3.4.1 (https://www.qgis.org).

Results of assessing MaxEnt model performances showed that the model performed well based on AUC and TSS metrics (AUC = 0.938 and TSS = 0.816). Results showed that the *M. thukuhar*/*cannicci* species complex suitable habitats are located in the following six marine biogeographic realms; Temperate Southern Africa, Western Indo-Pacific, Central Indo-Pacific, Eastern Indo-Pacific, Temperate Australasia and Temperate Northern Pacific (Fig. 2). Largest and smallest suitable habitats are located in Central Indo-Pacific and Temperate Southern Africa respectively (Fig. 1).

### Variables importance

Based on MaxEnt results, sea surface temperature with 38.1% contribution followed by nitrate (26.4%), and dissolved oxygen (14.6%) are the most important drivers of the complex distribution (Table 1). The probability of the complex presence is positively correlated with sea surface temperature and as temperature increased the area became more suitable for the complex and habitat suitability reached a peak at 30 °C.

**Table 1:** Variable contribution in habitat suitability model of the *Metopograpsus thukuhar*/*cannicci* species complex*.*

Variable	Contribution (%) to MaxEnt model
Mean sea surface temperature	38.1
Nitrate	26.4
Dissolved oxygen	14.6
Calcite	5.7
Tide average	4.8
Primary productivity	3.1
Saturated O_2_	2.6
Salinity	1.7
Range sea surface temperature	1.8
pH	1.2

### Marine protected areas coverage

Results of estimating marine protected areas coverage across the complex range showed that 12.5% of the species suitable habitats located within marine protected areas (Fig. 2). The highest proportion of protected suitable habitats (22.9%) are located in Western Indo-Pacific realm while the lowest proportion of protected suitable habitats (1.38%) were located in Central Indo-Pacific realm (Table 2). No protected suitable habitat was found for the complex in Temperate Southern Africa realm.

**Table 2:** Estimates of suitable habitats, protected suitable habitats and percentage of suitable protected habitats of the *Metopograpsus thukuhar*/*cannicci* species complex within the five marine biogeographic realms.

Marine biogeographic realms	Suitable area (km^2^)	Protected suitable habitat (km^2^)	Percentage of suitable protected habitat
Western Indo-Pacific	3,274,289	55,985	22.9
Central Indo-Pacific	7,783,576	49,456	1.38
Eastern Indo-Pacific	1,285,675	135,799	4
Temperate Northern Pacific	736,912	21,066	7.4
Temperate Southern Africa	24,769	0	0
Temperate Australasia	106,824	44,513	2.94

## Discussion

Mangrove forests are threatened by several anthropogenic activities^1,12,20^. But to conserve mangrove ecosystems it is necessary to save species that play important role in mangrove survival like the mangrove crabs^21^. The present study is the first to determine the habitat suitability of the *M. thukuhar*/*cannicci* species complex across its global distribution range and estimate protected area coverage for the complex suitable habitats within marine biogeographic realms. We found that the highest proportion of the complex’ suitable habitat is located in the Central Indo-Pacific realm hence this realm has high priority for the complex and consequently mangrove forest conservation.

Results showed that sea surface temperature was the most important determinant of the complex global distribution. Most crabs avoid temperatures above 29 °C, this is showed by the response of the complex to the sea surface temperature. Habitat suitability increase for the complex by an increase in temperature until 30 °C. Our results are in line with previous studies that identified sea surface temperature as a major determinant of distribution of marine organisms^50,51^ as well as different marine crustacean species^23,52^. Nutrients like calcite and nitrate are important determinants of mangrove crabs’ richness^23^. Our results confirm the importance of nutrients for a mangrove crab species complex. We found that saturated O_2_, range sea surface temperature, salinity, primary productivity, and pH play little role in shaping this complex distribution supporting previous findings that these variables have little contribution in predicting richness of mangrove crabs^23^.

Climate change is known as a major driver of biodiversity loss worldwide^18,53–55^. Previous studies have shown that marine species will be negatively affected by climate change^53,56–58^. Based on MaxEnt results, sea surface temperature turned out to be the most important determinants of the complex distribution but under climate change sea surface temperature will change^56,57^. Thus, the complex distribution will likely vary under changing climate. In addition, the species are tree-climbers in mangroves^47^ and strongly depends on mangroves but studies are showing that climate change is negatively influencing mangroves, making the species more vulnerable to climate change^12,14^. To be able to set proper programs for the complex conservation under climate change, it is necessary to identify the complex’ future suitable habitats and propose those areas as target areas for new marine protected areas.

Marine biodiversity is being lost at an increasing rate due to climate change, urban and industrial developments, overfishing and pollutions^53,58^. In this situation, marine protected areas are currently the most effective tools for conservation and management of marine ecosystems^59,60^. At the moment not only a small proportion of marine ecosystems are highly protected (2.7%) but their effectiveness is in doubt due to increasing anthropogenic effects^61,62^. SDMs are very informative tools to assess the effectiveness of marine protected areas in conservation of marine biodiversity and propose new protected areas as previously shown for marine species^63–65^. To our knowledge the effectiveness of marine protected areas in the conservation of mangrove crabs has been rarely assessed. Here we quantified protected areas coverage for a mangrove crab’ suitable habitats and showed that low level (12.5%) of the complex suitable range is legally protected. The percentage of protected suitable habitats of the complex varies among the realms from zero in Temperate Southern Africa to 22.9 in Western Indo-Pacific. Despite a large proportion of the complex suitable habitats being located within the Central Indo-Pacific realm, only 1.38% of them are protected. Thus, this realm has high priority for future development of marine protected areas for conservation of mangrove crabs. This shows that it is important to quantify protected areas coverage for every species within each biogeographic realm.

One key application of SDMs is to identify potential areas for species distribution and determine patches that are suitable but un-sampled^27,66^. Our MaxEnt model identified suitable patches with no distribution record for the *M. thukuhar*/*cannicci* species complex in particularly in the Tropical Atlantic and Tropical Eastern Pacific realms. We recommend those patches as suitable target areas for further field sampling^36,67^ to identifying all populations of the complex across its potential distribution range. It should be also noted that correlative SDMs are static so that they do not consider species dispersal barriers when predicting suitable range for a target species^27^. In fact, they identify areas with high suitability for a target species only by considering those environmental variables which were used in the model^27^. Thus, our model identified some suitable patches in which the species cannot be present due to ecological conditions or dispersal barriers particularly in Temperate Northern Pacific in west of North America.

## Conclusions

We identified the most suitable habitat of *M. thukuhar*/*cannicci* species complex and determined the most influential driver of the complex distribution. In addition, suitable but not protected habitats of the complex identified and were proposed as important target for future marine protected areas development especially in the Central Indo-Pacific realm. Results of this research increased our ecological knowledge of mangrove crabs and can be used to safeguard these ecologically important mangrove-dwelling crabs. Marine ecosystems are home to millions of species^16^ of which many of them are threatened with extinction due to several threats like climate change, pollution, overfishing, habitat destruction, land use changes and urban and agricultural development along coastlines^68^. In this regard, SDMs can be used to map species distribution over vast and remote areas of marine ecosystems^69,70^ and facilitate marine biodiversity conservation^69,71,72^.

## Materials and methods

### Occurrence data

**Figure 3:**
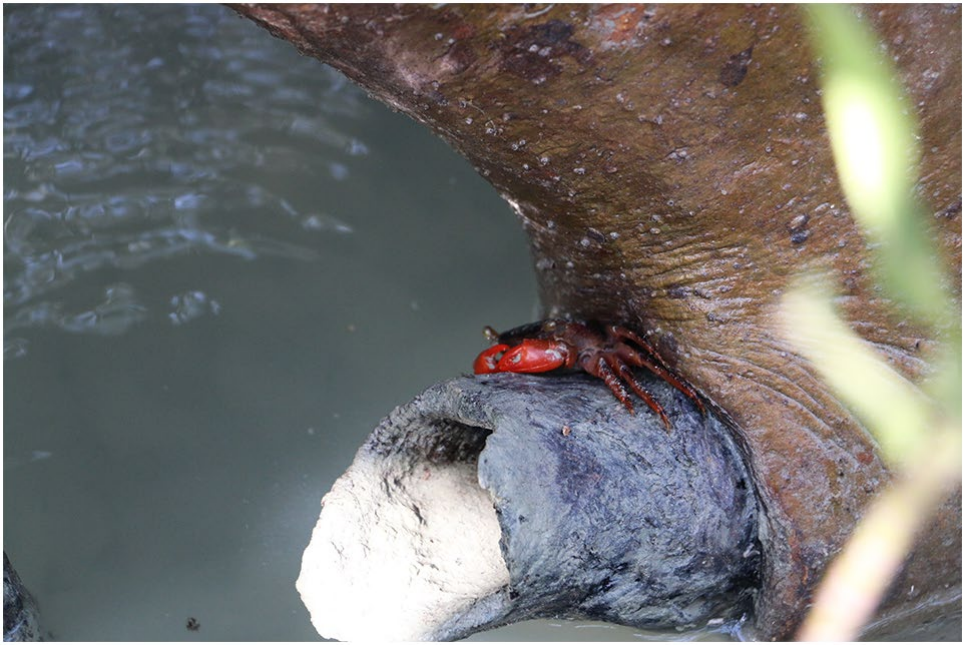
Photograph of *Metopograpsus cannicci* in its natural habitats in the Gulf of Oman. Photo by Reza Naderloo.

**Figure 4:**
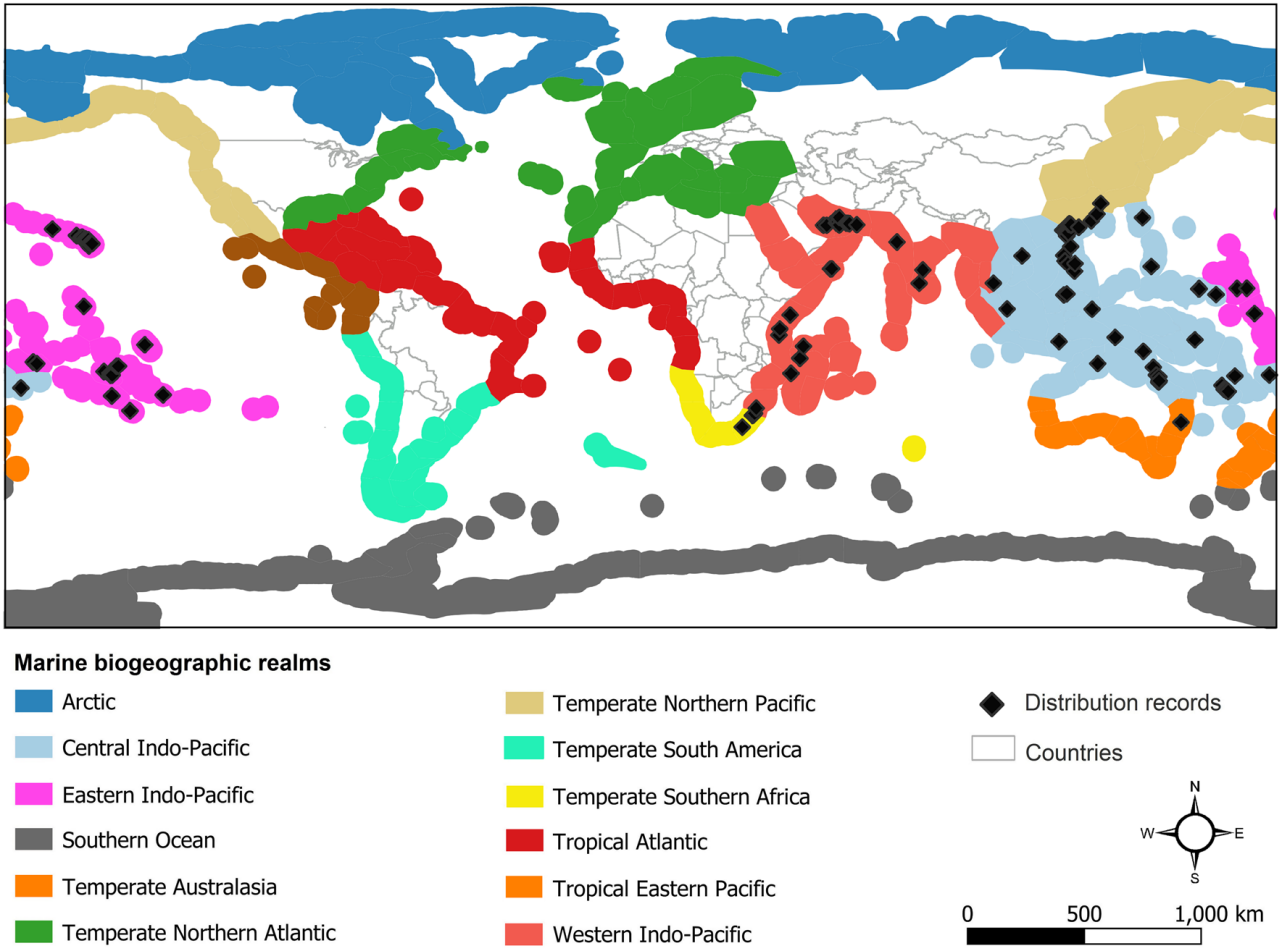
Global distribution of the *Metopograpsus thukuhar*/*cannicci* species complex and marine biogeographic realms^74^. The map was generated using QGIS 3.4.1 (https://www.qgis.org).

Distribution records of the *M. thukuhar*/*cannicci* species complex were gathered from different sources as follows: fieldworks (Fig. 3), online databases like the Ocean Biogeographic Information System (OBIS) and the Global Biodiversity Information Facility (GBIF) and published papers, books and atlases^46,47,73^. After gathering distribution records from different sources, duplicates and localities without coordinates were removed. In addition, distribution records were thinned to match with environmental layers’ resolution. In total, 235 distribution records were collected but after filtering them, 167 points remained and were used in distribution modeling (Fig. 4).

### Marine environmental predictors

To map global distribution of the *M. thukuhar*/*cannicci* species complex we used 10 environmental variables quantifying climatic, biological and geophysical conditions across the species distribution range^75^. Following variables were included in habitat suitability modeling; mean sea surface temperature (°C), tide average (m), salinity (PSS), primary productivity (mg C m^−2^ day^−1^ cell^−1^), dissolved oxygen (ml l^−1^), saturated oxygen (ml l^−1^), nitrate (μmol l^−1^), calcite (mol m^−3^), pH, and range sea surface temperature (°C). The environmental variables were obtained from the Global Marine Environment Datasets (GMED^75^) at 5 arc min spatial resolution. These variables are important in shaping marine crabs’ distribution^23,28,35,76–78^. To avoid using highly correlated variables (r ≥ 0.75) together in ecological niche modeling, a Pearson correlation test was performed (Supplementary Table S1).

### Habitat suitability modeling

In this study we used Maximum Entropy Modeling approach (MaxEnt) which is one of the best methods among many algorithms for modeling species distribution patterns^24,79^. This method only needs presence data from target species and is very effective even when distribution data is scarce^37^. Habitat suitability modeling was performed in sdm package^80^ in R environment^81^. To assess model performance, we used a split-sample approach (75% training data and 25% evaluation data) with 10 repetitions. Performance was measured using ROC AUC curves^27,82,83^ and True Skill Statistics (TSS) values^27^. AUC values range from 0 to 1, values close to 0.5 suggest that the model has no predictive ability while values close to 1 show perfect predictive ability^27^. TSS values range from − 1 to + 1, where + 1 indicates perfect performance and value of zero meaning random predictions.

### Marine protected areas coverage and marine biogeographic realms

To determine the representation level of suitable habitats of the complex inside marine protected areas, the continuous habitat suitability map was converted into binary suitable-unsuitable map. The 10 percentile training presence threshold was used to convert continuous map into binary^24,84^. Then, the binary habitat suitability model was overlaid on the marine protected areas layer. Finally, the area of suitable habitat inside the marine protected areas was calculated using the raster package in R^85^. Marine protected areas data obtained from Protected Planet (www.protectedplanet.net)^86. ^We calculated areas of suitable habitats within each of the following six marine biogeographic realms; Temperate Southern Africa, Western Indo-Pacific, Central Indo-Pacific, Eastern Indo-Pacific, Temperate Australasia and Temperate Northern Pacific^74^ that *M*. *thukuhar/cannicci* is present.

## Supplementary Information

Supplementary Information.

## Data Availability

All data needed to evaluate the conclusions in the paper are present in the paper and/or the Supplementary Materials, or the references cited here within.
