# Supplementary material for: Global habitat suitability modeling reveals insufficient habitat protection for mangrove crabs
Source: Sci Rep. 2022 Dec 15;12:21713. doi: 10.1038/s41598-022-26226-7 (PMC9755133; doi:10.1038/s41598-022-26226-7)
Supplement: Supplementary file 1 — Supplementary Information. [file 41598_2022_26226_MOESM1_ESM.pdf]

Supplementary Information

Global habitat suitability modeling reveals insufficient habitat protection for mangrove crabs

Masoud Yousefi and Reza Naderloo

School of Biology, College of Science, University of Tehran, 14155-6455 Tehran, Iran.

Table S1. Result of Pearson's correlation coefficient test.

Variables	Mean sea surface temperature	Calcite	Dissolved oxygen	Nitrate	PH	Primary productivity	Salinity	Saturated O2	Range sea surface temperature	Tide average
Mean sea surface temperature	1	0.551	-0.771	-0.616	0.423	-0.075	0.384	0.466	-0.247	-0.129
Calcite	1	1	-0.656	-0.573	0.343	-0.292	0.333	0.332	-0.125	-0.077

Dissolved oxygen	1	1	1	0.830	-0.417	-0.026	-0.413	-0.518	0.043	0.049
Nitrate	1	1	1	1	-0.476	-0.171	-0.216	-0.696	-0.256	0.054
pH	1	1	1	1	1	-0.088	0.205	0.374	0.109	0.012
Primary productivity	1	1	1	1	1	1	-0.296	0.191	0.421	0.169
Salinity	1	1	1	1	1	1	1	0.136	-0.275	-0.030
Saturated oxygen	1	1	1	1	1	1	1	1	0.363	-0.214
Range sea surface temperature	1	1	1	1	1	1	1	1	1	-0.064
Tide average	1	1	1	1	1	1	1	1	1	1
